# High Modulus Epoxy/GO-PANI Self-Healing Materials Without Catalyst by Molecular Engineering and Nanocomposite Fabrication

**DOI:** 10.3390/polym16223173

**Published:** 2024-11-14

**Authors:** Geonwoo Kim, Cigdem Caglayan, Gun Jin Yun

**Affiliations:** 1 Department of Aerospace Engineering, Seoul National University, Seoul 08826, Republic of Korea; rjsgun0805@snu.ac.kr (G.K.); caglayanci@snu.ac.kr (C.C.); 2 Institute of Advanced Aerospace Engineering Technology, Seoul National University, Seoul 08826, Republic of Korea

**Keywords:** epoxy, self-healing materials, nanocomposite, GO-PANI

## Abstract

Nowadays, self-healing materials have been studied actively in electronics, soft robotics, aerospace, and automobiles because they can prolong the life span of the materials. However, overcoming the trade-off relationship between mechanical properties and self-healing performance is challenging. Herein, graphene oxide-polyaniline (GO-PANI) filler was introduced to overcome this challenge because GO has a highly excellent modulus, and nitrogen atoms in PANI can endow a self-healing ability through hydrogen bonds. Aside from the hydrogen bond in PANI, the hydrogen bond in the carbonyl group and the disulfide exchange bond in the epoxy matrix also helped the materials heal efficiently. Therefore, the modulus of SV-GPN1 (Self-healing Vitrimer-GO-PANI1) reached 770 MPa, and a 65.0% healing efficiency was demonstrated. The modulus and self-healing efficiency were enhanced after adding GO-PANI filler. The self-healing ability, however, deteriorated when adding more GO-PANI filler because it hindered the collision between the molecules. Meanwhile, SV-GPN1 was excellent in reproducibility, which was proven by the experiment that 16.50 mm thick SV-GPN1 also displayed a self-healing ability. Thus, SV-GPN1 can be applied to structural materials in industries like aerospace because of its self-healing ability, excellent modulus, and reproducibility.

## 1. Introduction

Self-healing materials are in high demand to progress to a sustainable society [1,2]. Because self-healing materials can be applied in a variety of fields like electronics [3,4,5], soft robots [6,7], protective coatings [8,9], aerospace crafts [10,11], and automobiles [12,13], recent researchers have jumped into advancement in various self-healing materials. Despite enormous research, it is still challenging to develop self-healing materials that simultaneously possess a healing ability and outstanding mechanical properties [14], like high toughness, Young’s modulus, and tensile strength with healing performance.

Many efforts have been made to overcome this trade-off relationship problem, especially with thermoplastic polyurethanes or thermoplastic polyureas (TPUs) [15,16,17,18,19,20,21,22,23,24]. For example, Li et al. developed self-healing materials with high strength and toughness based on molecular engineering via spider silk-like structured TPUs [23]. Zhu and colleagues reported self-healing TPUs reinforced with graphene oxide (GO) to endow multiple hydrogen bonds to improve the mechanical properties [24]. Li and co-workers studied self-reinforcing TPUs through strain-induced crystallization to overcome the trade-off relationship between mechanical properties and healing ability [22]. Also, Eom et al. synthesized mechano-responsive hydrogen bonding array TPUs that are both healable and strong simultaneously [15].

However, despite these desperate efforts, TPUs have several limitations to be employed in aerospace industries. Above all, as TPUs are too elastic, they are inappropriate for field application [25]. In aerospace fields, deformation should be minimized to keep stability, and materials should be utilized as little as possible to reduce weight [26]. To satisfy these two requirements simultaneously, high-modulus materials should be developed. Most self-healing TPUs, however, have Young’s modulus of less than 100 MPa even though they have high strength values [15,16,17,18,19,20,21,22,23,24]. That is why TPU materials, which have low Young’s modulus, are not proper for aerospace industry. Accordingly, TPUs cannot be candidates for aerospace structural materials and other material types should be developed to overcome these disadvantages.

Unlike TPUs, epoxy materials are utilized actively in aerospace fields [27] instead of TPUs due to their high mechanical properties, including strength, modulus, and everything. That is because epoxy materials, composed of three-dimensional network structures, represent high material properties, including mechanical properties [28]. Therefore, it is highly recommended that self-healing materials made from epoxy be developed for the aerospace industry [29], and it will lead to the result that self-healing materials can be applied in aerospace. Despite the superior properties of epoxy polymers, they have low chain mobility and diffusibility, which requires high self-healing performance [15]. Due to these requirements, epoxy self-healing materials also cannot possess high stiffness [30,31,32,33,34,35,36,37,38,39,40,41]. Many researchers have tried to develop self-healing materials that have high modulus, but they provided external pressure to heal the self-healing materials [42,43,44,45,46,47,48,49]. This method is not only practical but also feasible in actual application.

Also, epoxy materials have a variety of applications due to their superior properties, which include chemical resistance, heat resistance, and ease of processing. Therefore, lots of fields like electronics, construction, and automobiles employ epoxy materials [50]. Due to the many advantages and applicability, epoxy materials are now being actively studied, including their anti-flammability [51,52], biodegradable epoxy [53], high thermal conductivity [54], and low dielectric [55] properties. That is also one of the reasons epoxy self-healing materials should be developed. Through epoxy self-healing materials, self-healing materials can be applied in many fields, advancing the commercialization of self-healing materials.

In our previous work [56], self-healing epoxy material was fabricated by molecular engineering. We found out which molecular design leads to optimized self-healing performance. Despite that, there is a problem in that the mechanical properties were not high enough to be applied to structural materials, even though they were epoxy materials. Meanwhile, graphene-oxide-modified with polyaniline (GO-PANI) is one of the most used nanofillers in epoxy nanocomposite materials. There are several reasons why GO-PANI is widely used in the nanocomposite field. First of all, the polymer chain prevents GO from aggregating. This characteristic makes nanofiller well-dispersed in the composite matrix. Next, GO-PANI can be used as reinforcement because graphene oxide (GO) has excellent mechanical properties [57,58,59,60,61]. Even though GO-PANI can be a good candidate for nanofillers, it has not been employed for self-healing nanocomposites [29]. Therefore, GO-PANI was utilized to synthesize self-healing nanocomposites in this study to make high-modulus healable materials.

Herein, GO-PANI-reinforced self-healing materials were synthesized to obtain high-modulus self-healing materials with improved healing efficiency. SV (Self-healing Vitrimer)-Neat, healable epoxy materials synthesized by our group were employed as an epoxy resin matrix, and GO-PANI was used as a nanofiller. A high-modulus self-healing composite can be obtained through a GO-PANI filler, and the self-healing performance did not deteriorate, even though the modulus was improved. This can be realized by overcoming the trade-off between mechanical properties and the healing ability in self-healing materials. To the best of the author’s knowledge, the modulus value has the highest value compared to other studies about self-healing materials without any external pressure. This result originated from GO-PANI properties, which helped improve both mechanical properties and self-healing efficiency of self-healing materials. Due to the exceptional mechanical properties of GO, GO-PANI filler made modulus improved, and due to the lots of hydrogens attached to nitrogen in PANI, the hydrogens can interact with nitrogen atoms between the fractured parts, which results in improved self-healing behavior [62]. Therefore, adding a GO-PANI filler can help overcome the trade-off relationship between mechanical properties and healing ability, which leads to high-modulus self-healing materials. By employing these features of PANI, SV-GPN1 (Self-healing Vitrimer-GO-PANI1) has a 770 MPa modulus value, the highest modulus among self-healing materials to the best of our knowledge. Moreover, the reproducibility was also excellent because the materials were synthesized using molecular design to represent high self-healing ability. Also, various concentrations of GO-PANI were conducted to confirm the profound effect of GO-PANI in this study.

## 2. Materials and Methods

### 2.1. Materials

Bisphenol A diglycidyl ether (DGEBA), polypropylene glycol diglycidyl ether (PPGDG), and ammonium persulfate were supplied from Sigma-Aldrich (St. Louis, MO, USA). 2-Aminophenyl disulfide (2-AFD) was provided from Aladdin (Shanghai, China). Diglycidyl 1,2-cyclohexane-dicarboxylate (DGCHD) was purchased from TCI (Tokyo, Japan). Graphite powder was supplied from Kanto Chemical (Tokyo, Japan). Junsei Chemical (Tokyo, Japan) provided potassium permanganate (KMnO_4_). Supplies involving 30% Hydrogen peroxide, 35% hydrochloric acid (HCl), sodium nitrate (NaNO_3_), and aniline were supplied by Samchun Chemicals (Pyeongtaek, Republic of Korea). All chemical reagents were used as received. Epoxy resin and hardener chemical structures are depicted in our previous work [56] (Figure S1).

### 2.2. Preparation GO and GO-PANI

To synthesize GO-PANI, GO was synthesized through the Hummers method before fabricating GO-PANI. The GO synthesis process is as follows. The sulfuric acid was poured into the beaker containing graphite powder and sodium nitrate (NaNO_3_) in an ice bath, and then the mixture was stirred for 15 min. Next, potassium permanganate (KMnO_4_) (3 mg) was added at room temperature and stirred for 30 min, and then the mixture was stirred for 7 h at 35 °C. Potassium permanganate (KMnO_4_) (3 g) was added and stirred overnight. The mixture was left at room temperature, and cold water (133 mL) and hydrogen peroxide (H_2_O_2_) (3 mL) were poured into the mixture, and it reacted for 90 min. Finally, GO was obtained by centrifuge and then washed with ethanol, 5% HCl, and water thrice. The GO was dried at 80 °C for 36 h.

**Figure 1:**
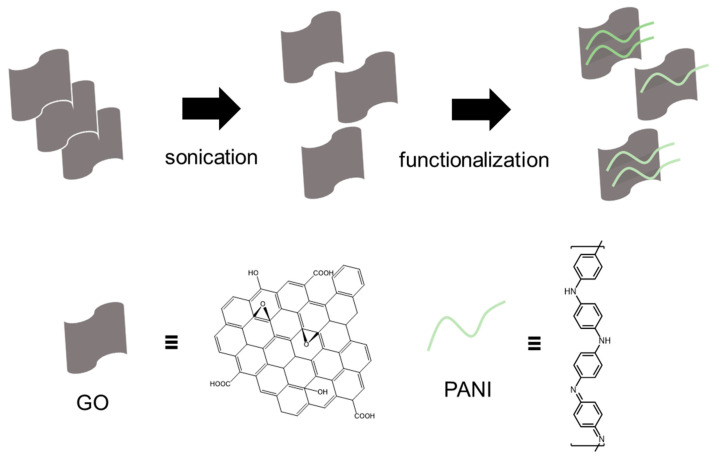
Illustration of synthesis route of GO-PANI. GO was sonicated and functionalized with PANI.

The obtained GO was functionalized with polyaniline (PANI) through a typical in-situ oxidative polymerization with ammonium persulfate. The GO-PANI synthesis process is as follows. First, the obtained GO was dispersed in water (15 mL) by ultrasonication, and aniline (0.15 g) and ammonium persulfate (0.09 g) were added in 1 M HCl (7.5 mL). The HCl solution and GO dispersed water were mixed and stirred for 24 h at room temperature. Then, the mixture was centrifuged and washed with DI water three times. The synthesis process and GO-PANI structure are described in Figure 1.

### 2.3. Characterization GO and GO-PANI

Fourier Transform Infrared spectroscopy (FTIR) spectra were analyzed by Vertex80V spectrometer from BRUKER (Preston, Australia) to investigate the chemical structure of GO and GO-PANI. GO and GO-PANI were made into pellets to analyze the chemical structures. All samples were scanned 32 times at a resolution of 4 cm^−1^, and spectra were 400–4000 cm^−1^. GO and GO-PANI thermal stability was analyzed by Thermogravimetric analysis (TGA) experiments with Discovery TGA from TA Instrument (New Castle, DE, USA) under air at a heating rate of 10 °C/min from 25 to 600 °C, and TGA investigated the degradation temperature. Raman spectroscopy was also employed with the DXR2xi model to analyze the GO and GO-PANI structures. X-ray Diffraction (XRD) experiments were introduced to confirm the crystalline structure of GO and GO-PANI. The 2θ range was 5–60 degrees, the scan speed was 1 s/step, and the wavelength range was 1–1.5418 Å using Cu kα. FE-SEM (Zeiss Sigma, Carl Zeiss, UK) was used to observe the morphological structure of nanofiller, GO, and GO-PANI.

### 2.4. Synthesis of Self-Healing Epoxy/GO-PANI Nanocomposite

**Figure 2:**
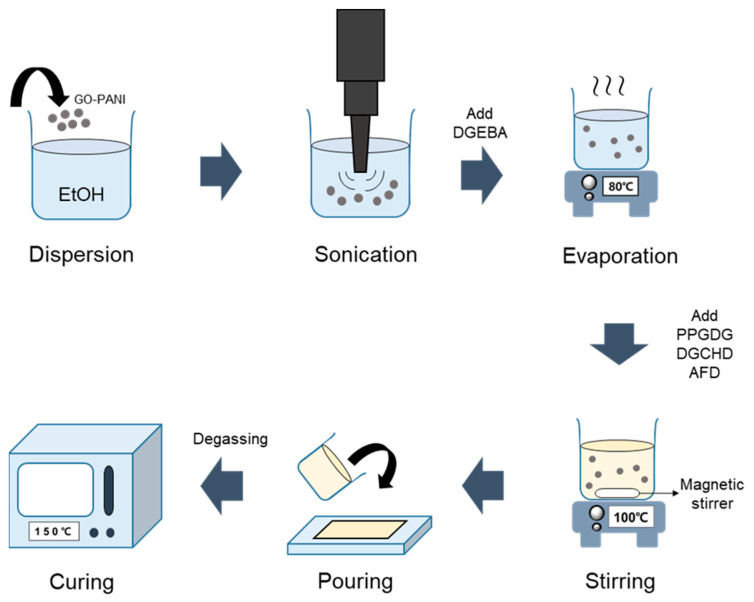
Diagram of preparation process of SV/GO-PANI nanocomposites.

The SV-Neat synthesis process is depicted in our previous study [56]. DGEBA (~170.2 EEW, 10,000–12,000 mPa·s), PPGDG (~320 EEW, 90–110 mPa·s), DGCHD (~142.15 EEW, 500–1000 mPa·s), and 2-AFD were stirred at 100 °C for 20 min on a magnetic stirrer, and then the mixed liquid was poured into a silicone mold. Next, the mixture was put in a vacuum oven at 80 °C for three hours and reacted at 150 °C for 15 h in an oven. The amount of monomer was as follows: DGEBA 3 g, PPGDG 2.82 g, DGCHD 2.52 g, and 2-AFD 3.62 g. The molar ratio is shown in Table 1. Epoxy/GO-PANI nanocomposite was fabricated through ultrasonication. First, the GO-PANI weight was measured, and GO-PANI was dispersed in ethanol. After dispersion, DGEBA (3 g) was added to the dispersed GO-PANI. Then, the ethanol solvent was evaporated at 80 °C overnight. After evaporation, PPGDG, DGCHD, and 2-AFD were added, stirred at 100 °C for 20 min, and poured into a silicon mold. The mixture was degassed at 80 °C for 3 h and cured for 15 h at 150 °C. The preparation process is shown in Figure 2, and the amount of resin and filler is tabulated in Table 2. SV-GPN0.5, SV-GPN1 and SV-GPN2 contains 0.05 wt%, 0.1 wt% and 0.2 wt% GO-PANI, respectively.

### 2.5. Characterization of Self-Healing Epoxy/GO-PANI Nanocomposite

Differential scanning calorimetry (DSC) experiments were executed utilizing Discovery DSC from the TA instrument. The temperature was in the range of −30 °C to 150 °C for GO-PANI nanocomposites and 0 °C to 150 °C for SV under nitrogen. The reason for the broader temperature range for nanocomposite than that for SV was to obtain clear T_g_ values. The heating ramp rate was set to 10 °C/min.

Thermogravimetric analysis (TGA) analysis condition was identical to GO and GO-PANI analysis.

The FTIR analysis condition was also identical to the GO and GO-PANI characterizations. However, unlike GO and GO-PANI, the samples were not made into pellets but analyzed as they were in ATR mode.

### 2.6. Self-Healing Experiments

In this study, tensile tests were selected to estimate the self-healing efficiency, and a Psylotech micro-tensile testing machine was used to test the results. The thickness of the sample fabricated for tensile tests was 1.5 mm to 2 mm. The samples with 20 mm gauge length underwent a 200 μm/min strain rate at room temperature.

The self-healing process was performed in the following way. Firstly, the dumbbell-shaped specimen was cut in half. Then, the cut parts were stuck manually on the hot plate at 130 °C for a short while and left at 130 °C for 24 h without any specific stimulus like pressure, solvent, or light. The self-healing efficiency is measured as follows: the ratio of pristine strength to the healed strength as expressed in Equation (1).

(1) $$\eta =\frac{\sigma_{healed}}{\sigma_{original}}\times 100$$

## 3. Results and Discussion

**Figure 3:**
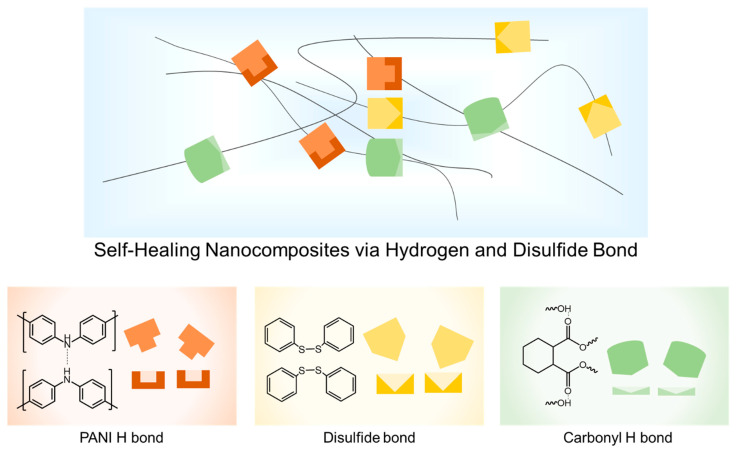
Schematic illustration of the molecular structure of the self-healing epoxy nanocomposites via hydrogen bond and disulfide exchange bond.

GO-PANI was dispersed in epoxy self-healing materials to synthesize self-healing nanocomposites with a high modulus. Then, three reversible bonds played a pivotal role in healing the materials: the hydrogen bond in PANI, the hydrogen bond in the carbonyl group (DGCHD), and the disulfide bond (2-AFD) (Figure 3). Accordingly, GO-PANI-reinforced epoxy materials were fabricated to utilize these reversible bonds. The fabrication process is as follows. Firstly, GO-PANI was synthesized by functionalizing GO with aniline. Then, GO-PANI epoxy nanocomposites were fabricated by dispersing GO-PANI in epoxy resin and hardener through the sonication method. Finally, the mixture was cured at 150 °C for 15 h.

### 3.1. Characterization of GO and GO-PANI

**Figure 4:**
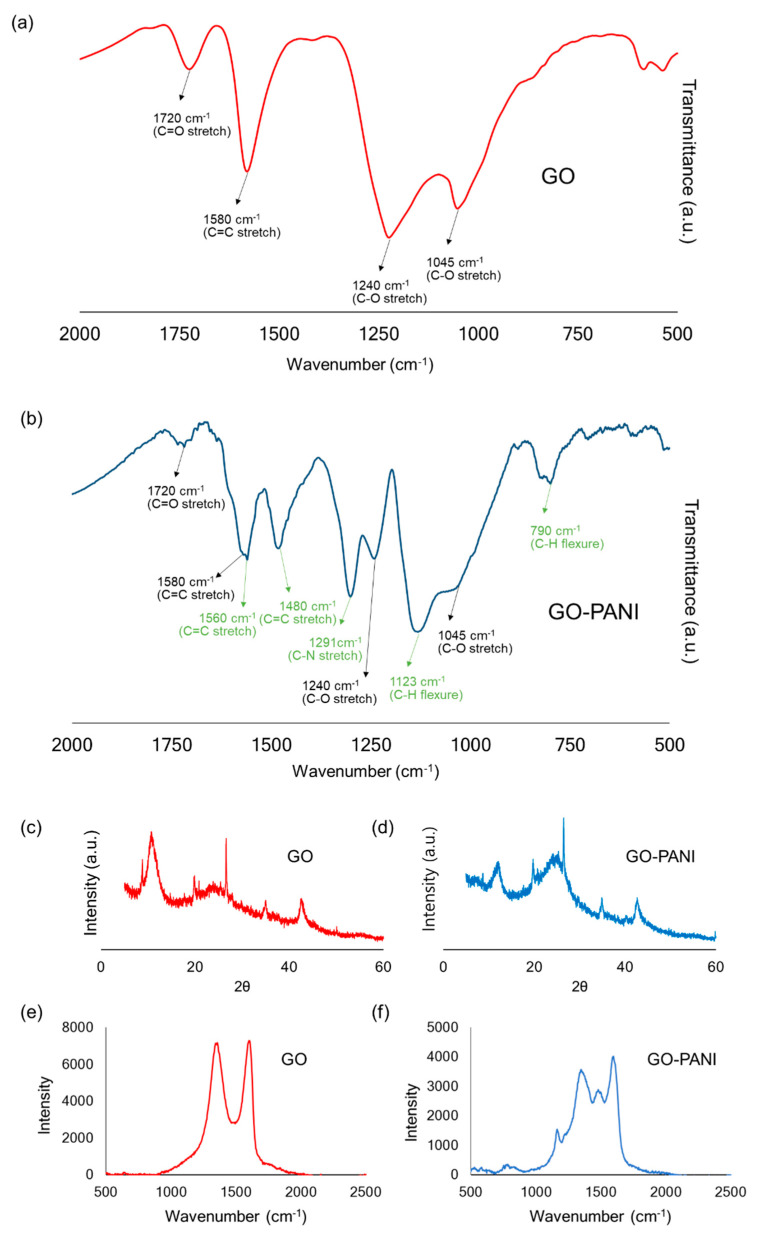

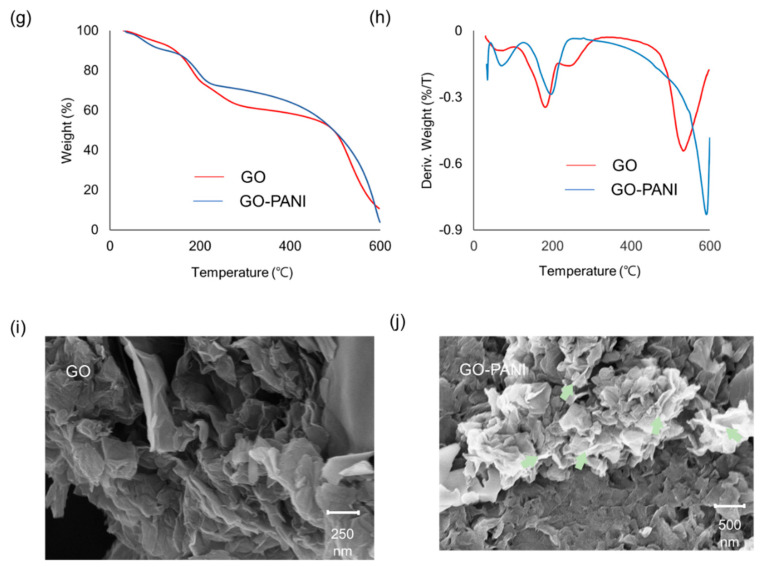
Characterization of GO and GO-PANI. FTIR spectra of (**a**) GO and (**b**) GO-PANI. The black arrows indicate the peak originating from GO, and the green arrows indicate the peak originating from PANI. XRD patterns of (**c**) GO and (**d**) GO-PANI. Raman spectra of (**e**) GO and (**f**) GO-PANI. (**g**) Weight reduction plots of GO and GO-PANI via TGA. (**h**) Derivative weight reduction profiles of GO and GO-PANI. (**i**) SEM image of (**i**) GO and (**j**) GO-PANI. Green arrows indicate the rigid rod-like polymer PANI.

GO was synthesized through the Hummers method, and we oxidized the graphite by employing KMnO_4_ and H_2_O_2_. After fabricating GO, polyaniline was functionalized on GO using ammonium persulfate. Then, the GO-PANI was prepared. FTIR, XRD, RAMAN, TGA, and SEM characterization experiments were executed to confirm PANI was functionalized to GO. Firstly, FTIR was performed to verify the functionalization of GO and discover the chemical structure (Figure 4a,b). Because the graphite was oxidized, some functional groups, such as carboxyl, hydroxyl, and epoxide, were found in GO. In the FTIR spectra, there was a C=O stretch in carbonyl (1720 cm^−1^), a C=C stretch in the aromatic ring (1580 cm^−1^), a C-O stretch in the epoxy group (1240 cm^−1^), and a C-O stretch in the alkoxy group (1045 cm^−1^) [63]. After GO was functionalized with PANI, a new peak appeared due to the bond in PANI. For example, GO-PANI showed a C=H stretch vibration in benzenoid (1560 cm^−1^), C=C stretch vibration in quinoid, C-N stretch in secondary aromatic amine (1291 cm^−1^), a C-H in-plane flexural vibration (1123 cm^−1^) and a C-H out-of-plane flexural vibration (790 cm^−1^) [64]. These results showed that PANI was attached to the GO sheet successfully.

XRD analysis was also performed to investigate the change in the crystalline structure of GO-PANI after functionalization. The GO pattern of XRD showed that the 2θ = 11° peak corresponded to 0.80 nm interlayer space, which originated from the oxidation of the graphite (Figure 4c). When the graphite, which had an sp^2^ orbital structure, was oxidized, the characteristic peak at 2θ = 11° appeared, which meant that oxygenated groups were formed on the graphite sheets. This is because some of the sp^2^ orbital from the graphite was changed into the sp^3^ orbital from the oxidized graphite, or a new crystal structure was developed [65]. Consequently, this peak showed that GO was synthesized successfully. The GO-PANI pattern also represented this peak, meaning the polymer chain was attached to a 2D GO plane with the remaining GO structure. Also, the 2θ = 25° broad peak appeared in the XRD pattern in GO-PANI, which did not exist in the GO pattern (Figure 4d). Because this broad peak was identical to the polyaniline XRD peak, it was suggested that polyaniline was attached to GO successfully. Apart from this peak, the peaks in GO and GO-PANI were very similar, which meant that the GO structure was almost identical before and after polyaniline was attached.

Next was the Raman analysis, which was used for the non-destructive analysis of carbon materials and was conducted to explore the graphite structure after GO functionalization. Figure 4e shows two characteristic bands, a D-band (around 1350 cm^−1^) and a G-band (around 1600 cm^−1^). The D-band was related to the density of defect structures like disorder, vacancies, and edge defects. On the other hand, the G-band appeared due to the normal graphite sheet from the sp^2^ orbital bond. By comparing the intensity of the D-band (I_D_) and G-band (I_G_), it was inferred how many defects were in the GO plane with the value of I_D_/I_G_. In the GO spectra, the I_D_/I_G_ value corresponded to 0.98. In GO-PANI (Figure 4f), there was a small increase of 1490 cm^−1^ and 1600 cm^−1^. These increases originated from Ar-NH and Ar-NHR in PANI. It indicates that there was PANI on the GO sheet [66]. Also, 1200 cm^−1^ was from the C-N stretch, and 811 cm^−1^ was from an amine deformation [67]. This Raman analysis suggests that GO was modified with PANI through a PANI functionalization process.

TGA experiments were carried out up to 600 °C to explore the thermal stability of GO and GO-PANI (Figure 4g,h). The temperature region was divided into four areas according to which molecule reacted to the heat, and the thermal degradation behavior was similar for GO and GO-PANI in these areas [68,69]. The first region corresponding to 100–120 °C was due to the evaporation of water adsorbed on the GO sheet. Although unrelated to the chemical reaction, a mass reduction in GO-PANI was faster than GO. This phenomenon can be explained by the fact that PANI was insoluble in water, which means PANI was repulsive to water. It made water removal easier in GO-PANI, and mass reduction in GO-PANI was faster. The second region (120–180 °C) was associated with the decomposition of oxygen-functional groups, such as OH functional groups. This functional group was less stable than other oxygen-functional groups, so it decomposed at lower temperatures. From this region, GO mass reduction was faster than GO-PANI. This result shows that GO had better thermal stability when it was functionalized with PANI. Thirdly, the 180–320 °C region showed the decomposition of more thermally stable oxygen-functional groups, like epoxide rings or carboxylic groups. Herein, GO-PANI thermal stability was more significant. Because the epoxide ring reacted with aniline, GO-PANI was expected to reduce the amount of epoxide ring. The reduction of the epoxide ring allowed GO-PANI to be more stable from heat. It also indicated that PANI was attached to GO by the functionalization. Also, this oxidation caused GO and GO-PANI to have relatively weak thermal stability because the C-O bond was weaker than the C=C double bond. Orbitals on graphite sheets were changed from sp^2^ into sp^3^, leading to weak thermal stability because π–π interactions diminished [70]. The last region was 420–600 °C, assigned to the pyrolysis of carbon in the GO sheet. From this region, GO-PANI mass reduction was remarkably rapid. It meant that the stability of the carbon skeleton in GO-PANI was a little inferior to that of GO [69]. After PANI functionalization, the overall thermal stability increased due to the PANI on the GO sheet. This also indicated that PANI functionalized GO was synthesized successfully.

The obtained SEM images of GO and GO-PANI are presented in Figure 4i,j. The surface morphology of GO had wavy sheet-like structures due to the sp^3^ orbital structures on the graphene sheet. These wrinkled structures increased the surface area of GO and confirmed that GO was synthesized successfully [71]. Although fold-like structures remained similar to GO structures in the GO-PANI morphology, some bumps appeared on the GO sheet. PANI represented rigid structures originating from 2D structures due to conjugation structures. The bumps emerged; the PANI chain showed a straight form, evidence that PANI functionalized GO. These characterization experiments showed that PANI was successfully attached to the GO sheet, so this GO-PANI would be employed to synthesize self-healing nanocomposites and confirm the effect of GO-PANI on nanocomposites properties.

### 3.2. Characterization of Resin and Nanocomposites

**Figure 5:**
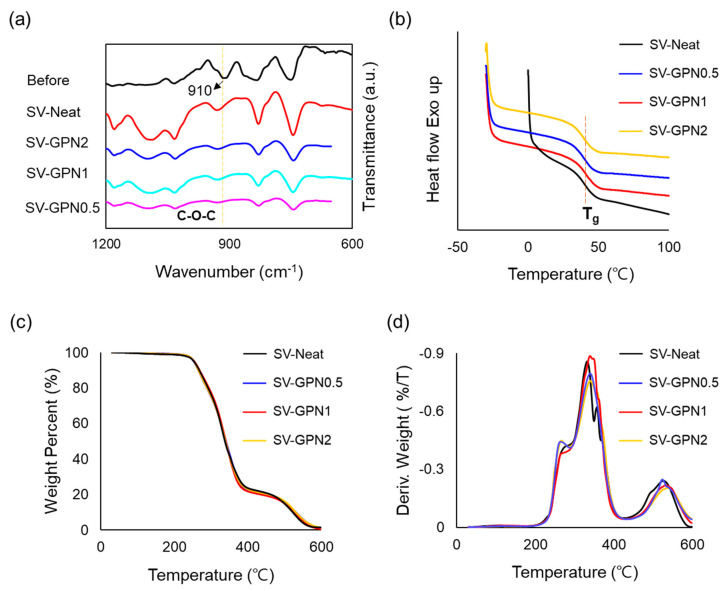
Characterization of neat epoxy resin and its nanocomposites. (**a**) FTIR spectra of uncured sample and SV-Neat, SV-GPN0.5, SV-GPN1, SV-GPN2. (**b**) DSC graph, TGA graphs corresponding to (**c**) weight reduction percentage and (**d**) derivative weight reduction of SV-Neat, SV-GPN0.5, SV-GPN1, and SV-GPN2.

With the synthesized GO-PANI, epoxy nanocomposites were fabricated through thermal curing. The well-dispersed GO-PANI can be confirmed, as shown in Figure S2. There were two reasons GO-PANI was well dispersed in the epoxy resin. Firstly, the oxygenated functional groups on the GO sheet, which included the epoxide ring, carboxylic acid, and hydroxyl group, dispersed GO-PANI well in the epoxy group. Also, the PANI chains prevented GO sheets from aggregating because the chains made it challenging for GO sheets to approach each other. That is why GO-PANI could be well-dispersed in epoxy resin. PANI dispersed well in the epoxy group in the resin, showing a hydrogen bond in polyaniline. Before investigating the self-healing and mechanical properties, several characterization experiments were conducted. TGA, DSC, and FTIR were undertaken to characterize the synthesized nanocomposites. First of all, a successful curing reaction was confirmed by FTIR experiments. As displayed in Figure 5a, the epoxide ring peak corresponding to 910 cm^−1^ was investigated to verify the synthesis state. While the epoxide ring peak was highly distinct in the uncured sample, FTIR spectra represented the epoxide ring peak, which was reduced after the curing process. These results demonstrated that the epoxide ring opening reaction occurred at a high temperature during the synthesis process, forming the epoxy network.

Moreover, the peak height was measured to explore the effect of GO-PANI filler on the degree of curing. The method of calculating the degree of curing was depicted in our previous work [56]. Briefly, the height of the epoxide ring peak (890 cm^−1^–950 cm^−1^) and ether bond peak (950 cm^−1^–1050 cm^−1^) was measured with absorbance peak (Figure S3). Then, the degree of curing peak was calculated by Equation (2).

(2) $$\alpha =\left[1-\frac{\left(h_{after}^{epoxy}/h_{after}^{ether}\right)}{\left(h_{before}^{epoxy}/h_{before}^{ether}\right)}\right]\times 100(\%)$$

where $h_{before}^{epoxy}$ and $h_{before}^{ether}$ are the heights of the epoxide ring and the ether bond before the curing reaction, respectively. $h_{after}^{epoxy}$ and $h_{after}^{ether}$ are the heights of the epoxide ring and the ether bond after the curing reaction, respectively. As tabulated in Table S1, the degree of curing value was similar to each other, which suggested that the curing reaction was not affected much by GO-PANI filler. These results were consistent with a study conducted by Nonahal and their colleagues [72]. The study is about the curing kinetics of GO-amine functionalized epoxy composite. Although carbon nanofiller hinders the epoxy-curing reaction, amine attached to GO assists in the curing reaction. It leads to the similar activation energy of pristine epoxy and epoxy nanocomposite reinforced with amine-functionalized GO. The authors explained that despite physical hindrance from GO, functionalizing amine facilitated epoxy-curing reactions because amine engaged in the reaction. Also, because the degree of curing was very similar for every sample, the degree of curing did not affect the self-healing properties. The difference in self-healing properties originated from GO-PANI filler.

Next, DSC analysis was conducted to confirm glass transition temperature (T_g_) values. T_g_ values can be obtained in DSC by comparing the reference and sample heat flow. Because T_g_ values are related to chain mobility and free volume in the molecules, molecular structures can be inferred through DSC experiments. Figure 5b shows the DSC analysis results. The results showed that every sample had a similar T_g_ value, corresponding to about 39 °C, regardless of the addition of GO-PANI nano reinforcement. The DSC results indicated that the epoxy network structures were resemblant, which meant there was no significant difference in the mobility of structures and the free volume after insertion of GO-PANI. These similar structures were attributed to two conflicting factors: curing hindrance from spatial occupancy of the nanofiller in the middle of network structures and curing assistance from amine functionalization originating from PANI. These results were consistent with FTIR results. Also, these results represented that molecular mobility, which generally affects the self-healing ability for self-healing materials, was not a factor that made a difference in self-healing efficiency for SV-Neat and other nanocomposites in this study.

Finally, TGA profiles were obtained to analyze the thermal stability of matrix resin and nanocomposites. Pristine resin and nanocomposites experienced the temperature ramping from 30 °C to 600 °C with a ten °C/min rate to observe the thermal degradation behavior. As shown in Figure 5c, thermal behavior was significantly similar regardless of any sample. The degradation temperature was organized for each sample (Table 3, Figure S4) to identify the thermal stability of the materials profoundly. T_5%_ and T_30%_ indicate the temperature at which the mass loss reached 5% and 30%, respectively, and were used to measure the static heat-resistance index (T_s_). The T_s_ value, obtained by calculating Equation (3), was employed to explore epoxy materials’ physical heat tolerance limit [73]. The T_s_ value not only represents the thermal stability of the materials but is also utilized as a reference for materials that can endure heat energy stably. Every sample showed about 140 °C T_s_ values, which means synthesized materials could tolerate heating up to 140 °C. Therefore, materials are not allowed to be exposed to a temperature over 140 °C to prevent thermal degradation. The T_s_ value is a good indicator for setting the healing temperature of the materials. That is why we set the healing temperature at 130 °C in order to avoid thermal aging.

Meanwhile, the maximum degradation rate temperature (T_dmax_) was also measured. While the T_dmax_ value of SV-Neat was 332 °C, the T_dmax_ values of others were about 339 °C, which suggested that GO-PANI affected the T_dmax_ values (Figure 5d). Although T_dmax_ is slightly different between pristine resin and nanocomposites, the overall thermal degradation behavior was almost identical.

T_s_ = 0.49 [T_5%_ + 0.6 (T_30%_ − T_5%_)] (3)

### 3.3. Self-Healing Experiments

Herein, the self-healing performance of the specimens was evaluated. Because every SV-GPN sample, including SV-GPN0.5, SV-GPN1, and SV-GPN2, possessed a disulfide bond, carbonyl H-bond, and PANI H-bond, these bonds could heal the samples. Our previous study [56] showed that disulfide bonds played a pivotal role in self-healing behavior, and a carbonyl H-bond also assisted materials in being healed. Meanwhile, the PANI H-bond also contributed to self-healing behavior in SV-GPN samples in this study, which will be proved below.

**Figure 6:**
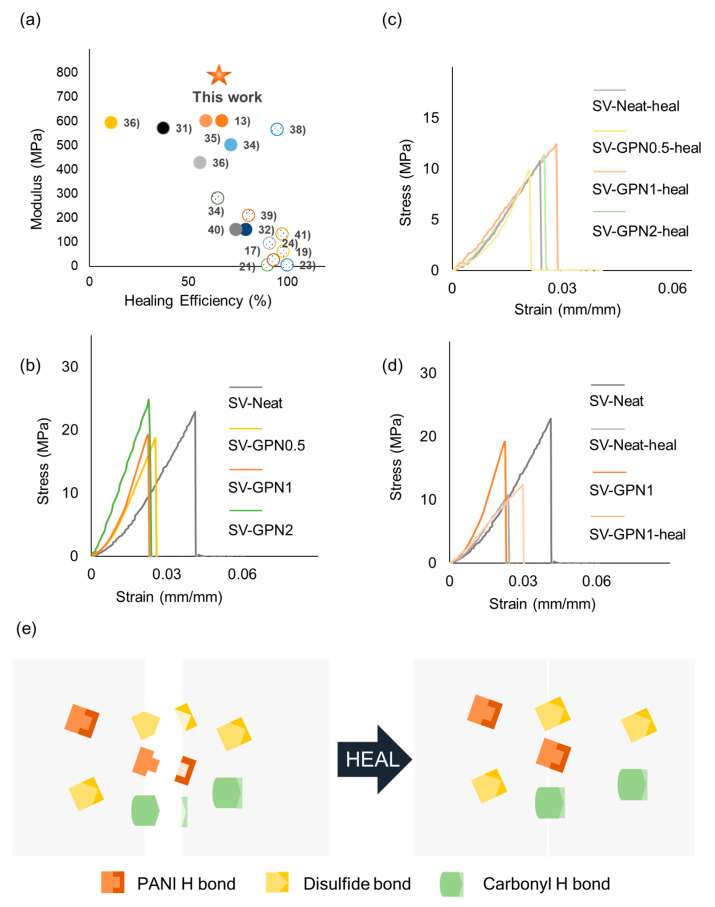
(**a**) Comparison of Young’s modulus and healing efficiency of SV-GPN1 and other literature related to healing materials without external pressure or force. (**b**) Stress-strain curve of pristine SV-Neat, SV-GPN0.5, SV-GPN1, and SV-GPN2. (**c**) Stress-strain curve of healed SV-Neat, SV-GPN0.5, SV-GPN1 and SV-GPN2. (**d**) Comparison of stress-strain curve of pristine SV-Neat and SV-GPN1, and healed SV-Neat and SV-GPN1. (**e**) Schematic illustration of the self-healing mechanism of epoxy self-healing materials reinforced with GO-PANI.

**Figure 7:**
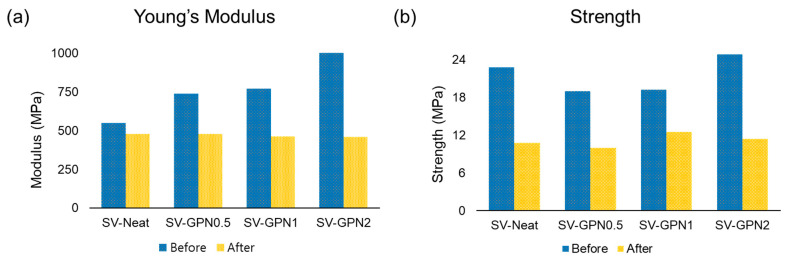
Mechanical properties of the samples. (**a**) Young’s Modulus and (**b**) strength of SV-Neat, SV-GPN0.5, SV-GPN1 and SV-GPN2 before and after healing.

Self-healing experiments were conducted via a tensile test to confirm the self-healing ability of the sample. The self-healing efficiency (η) was calculated by Equation (1). Briefly, the strength of the pristine and healed samples was compared to evaluate the self-healing ability of fabricated samples. First of all, for the sake of evaluation of self-healing performance, the mechanical properties of pristine samples were assessed (Figure 6b). The strength of SV-Neat, SV-GPN0.5, SV-GPN1, and SV-GPN2 was 22.8, 18.7, 19.2, and 24.8 MPa, respectively (Figure 7b). When GO-PANI was added, the strength was reduced slightly in SV-GPN0.5 and SV-GPN1. This reduction was related to the rigid PANI structure because the interface subjected to stress concentration made nanocomposites brittle compared to neat epoxy resin [74]. The interaction between GO-PANI and epoxy resin also affected mechanical properties. In DSC analysis, there were no significant differences between every sample, but the mobility decreased when nanofillers were added, which could lead to T_g_ value reduction. The repulsive interaction between resin and nanofillers can make T_g_ decrease. It meant that the interaction between GO-PANI and epoxy resin might not be good, resulting in embrittlement in nanocomposite samples. Despite that, GO-PANI can bear more load when GO-PANI is included more, which leads to a strength increase.

On the other hand, the modulus values rose when increasing the amount of GO-PANI. The modulus values for SV-Neat, SV-GPN0.5, SV-GPN1, and SV-GPN2 corresponded to 550, 740, 770, and 1050 MPa, respectively (Figure 7a). These results showed that the epoxy matrix transferred the external load to GO-PANI reinforcement, which meant that GO-PANI played a pivotal role in reinforcement. This can be inferred from the fact that when GO was added more, the modulus increased. That is because GO has a high modulus and PANI has a rigid-rod structure, so the load was transferred to GO-PANI, and the materials can bear more than neat samples. Accordingly, inserting GO-PANI filler in the epoxy resin is an excellent option to improve the materials’ modulus.

Next, the mechanical properties of healed samples were evaluated (Figure 6c). Self-healing experiments were conducted to measure the mechanical properties of healed samples. The samples were broken into two parts and then left at 130 °C. The healing temperature was set by referring to the T_s_ value from the TGA analysis. In other words, the healing temperature was set not to exceed the T_s_ value due to the thermal tolerance. The strength of the healed sample was 10.7, 9.9, 12.4, and 11.3 MPa, corresponding to SV-Neat, SV-GPN0.5, SV-GPN1, and SV-GPN2, respectively (Figure 7b). Then, the healing efficiency was 47.0%, 52.9%, 65.0%, and 45.6% for SV-Neat, SV-GPN0.5, SV-GPN1, and SV-GPN2, respectively. Surprisingly, when GO-PANI was added, the healing ability was improved even though the nanofiller hindered the collision between the epoxy polymer chain.

As we proved earlier in the FTIR and DSC analysis, GO-PANI inclusion did not affect the degree of curing and molecular mobility. Therefore, the improvement originated from the GO-PANI filler itself. Ge et al. [62] studied that polyaniline hydrogen bonds contributed to self-healing behavior. Thus, this improvement resulted from a hydrogen bond in PANI, which possesses nitrogen atoms. Nitrogen and hydrogen atoms can interact, forming hydrogen bonds and enhancing self-healing ability in nanocomposites. The PANI hydrogen bond can also explain why the self-healing ability increased when the GO-PANI ratio rose. When GO-PANI is increased, the hydrogen bond also increases.

FTIR spectra were obtained to confirm if there were hydrogen bonds in polyaniline. Because the 3290 cm^−1^ peak pointed out the hydrogen-bonded polyaniline [75], the peak appearance meant hydrogen bonds in nanocomposites. As shown in Figure S5, SV-Neat displayed a smooth line at 3290 cm^−1^. On the other hand, the spectra in nanocomposites showed a rough line due to the hydrogen bond generation. Even though the peak was not decisive because the amount of GO-PANI was considerably small, the peak was distinct enough to be observed.

Surprisingly, the enhanced self-healing properties were shown to improve mechanical properties because the self-healing behavior is affected by molecular mobility. However, the mechanical properties decrease when trying to strengthen molecular mobility to enhance self-healing performance. However, with the GO-PANI, self-healing efficiency can increase with enhancing modulus value. That is because the hydrogen bond in GO-PANI assisted self-healing behavior, and GO-PANI reinforcement enhanced the mechanical properties. Therefore, GO-PANI can be one of the candidates for overcoming the trade-off relationship between mechanical properties and self-healing behavior.

As shown in Figure 6a, SV-GPN1 showed the highest Young’s modulus among previous self-healing studies to the best of our knowledge. Note that this study considered the materials healable without external pressure as self-healing materials. Also, considering that SV-GPN has a network structure, the healing efficiency is not a low value, as shown in Figure 6a. Note that the solid circle indicates network structure polymer and the dotted circle indicates linear or thermoplastic polymer in Figure 6a. Because linear structure has higher mobility than network structure, it affects healing behavior, and linear polymer can be healed more efficiently, which leads to lower healing efficiency in network structure.

Because of the three reversible bonds indicating the H bond in polyaniline, disulfide bond, and H bond in the carbonyl group (Figure 6e), the nanocomposites are healable. As mentioned above, the disulfide bond played a crucial role in self-healing behavior, which can be inferred from the neat sample’s self-healing efficiency. The self-healing efficiency value for a neat sample was 47.0% even though GO-PANI did not exist in materials, which meant the disulfide bond played an essential role in self-healing. Although there were disulfide and carbonyl hydrogen bonds simultaneously, disulfide bonds contributed to the self-healing behavior more than carbonyl H bonds did. This can be confirmed by our previous work [56], in which it was proved that the disulfide bond was a critical factor in self-healing. However, it was not enough to make materials heal only with a disulfide bond, so a PANI hydrogen bond was also required to recover the sample. When GO-PANI was added, the self-healing efficiency increased from 47.0% to 65.0%. This indicated that the GO-PANI hydrogen bond also contributed to self-healing. The results also suggested that the PANI hydrogen bond assisted in making composites healed. However, the disulfide bond still played a significant role in self-healing because the efficiency increment ended up to 18.0%.

In summary, the disulfide bond played a significant role in self-healing in GO-PANI disulfide epoxy composites, and carbonyl and polyaniline hydrogen bonds helped materials to be healed. Self-healing efficiency decreased in SV-GPN2 samples because GO-PANI nanofiller hindered molecular collision. Epoxy molecules should meet each other for self-healing to occur. However, the probability of meeting each other decreased when there were many nanofillers in materials because nanofillers obstructed the epoxy molecular moving path. Even though the PANI hydrogen bond increased, self-healing efficiency decreased because disulfide was more critical for self-healing. The reduction of molecular collision led to a probability reduction in the disulfide bond exchange reaction. That is why the self-healing efficiency decreased.

Interestingly, the modulus of the nanocomposite decreased after healing, but the SV-Neat did not show a modulus decrease. Also, the modulus was almost identical to each other regardless of the samples (Figure 6c) and similar to the modulus of SV-Neat (Figure 6d). These results can be explained by the difference in the coefficient of thermal expansion (CTE). The resin expanded more than the nanofiller because epoxy resin and GO filler have different CTE values [76]. Then, a crack or gap appeared between the resin and filler due to the thermal expansion difference. Therefore, the epoxy resin cannot grip the nanofiller after exposure to heat, leading to a modulus decrease and a similar modulus value to the modulus of SV-Neat (Figure S6).

Meanwhile, a way to reduce embrittlement was suggested. Adding more PPGDG to the epoxy resin can relieve the embrittlement phenomenon. Figure S7 shows the stress-strain curve of the DSV-GPN1, ductile SV-GPN1, which includes 20% more PPGDG than SV-GPN1. Because the PPGDG chain was a linear aliphatic, epoxy molecules became softer. Therefore, the DSV-GPN1 was more malleable than SV-GPN1. Although the strength of the DSV-GPN1 was similar to SV-GPN1, the modulus value decreased to 555 MPa because the soft segment, PPGDG, was more contained compared to SV-GPN1. The value was kept at 66.0% for self-healing efficiency, almost identical to the SV-GPN1 sample.

**Figure 8:**
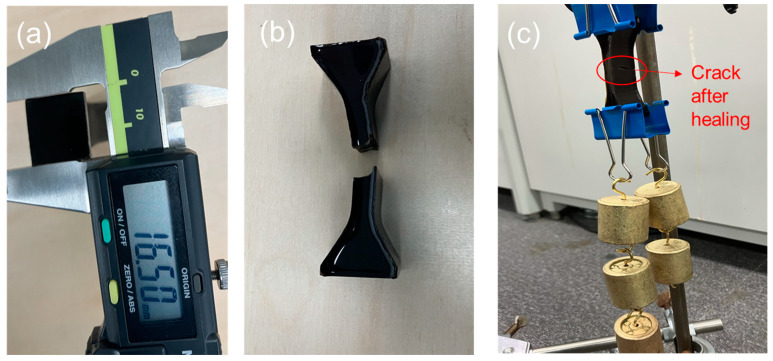
Photograph of (**a**) measurement of the thickness of SV-GPN1, (**b**) thick SV-GPN1 broken into two pieces and (**c**) load-bearing thick healed SV-GPN1 nanocomposite material.

Some self-healing materials have poor reproducibility due to the complicated conditions for healing processes. Sometimes, healing behavior occurs only for the specific shape or thin samples. Therefore, in this study, a thick SV-GPN1 sample was fabricated to verify the reproducibility of the sample. A sample as thick as 16.50 mm (Figure 8a) was selected to show the healing ability no matter the shape of the sample. SV-GPN1 was cut into two pieces (Figure 8b), and then the sample was exposed to 130 °C for several seconds. Then, several weights were hung and connected to the sample through a clip (Figure 8c). Though the sample has been broken once, it could bear the weight load. This experiment proved that the nanocomposites developed in this study represented excellent reproducibility.

## 4. Conclusions

In this paper, GO-PANI-reinforced epoxy self-healing materials were developed to improve mechanical properties and self-healing abilities simultaneously. Although two properties, mechanical properties, and self-healing abilities, have trade-off relations, GO-PANI could overcome this limitation by playing a role in reinforcement and reversible bonds. Therefore, the nanocomposite SV-GPN1 showed excellent modulus (770 MPa) compared to other self-healing materials because GO-PANI made the materials enhance mechanical properties, and it showed self-healing behavior due to the hydrogen bond in PANI. Aside from the hydrogen bond in PANI, disulfide and a hydrogen bond in the carbonyl group also contributed to the self-healing behavior. However, when GO-PANI was added to improve the mechanical properties and self-healing abilities, the self-healing performance fell because the filler hindered collision between the molecules.

Meanwhile, the nanocomposites developed in this study showed excellent reproducibility regardless of the shape and thickness. Therefore, SV-GPN1 can be considered a practical structural material like aerospace due to its self-healing ability, excellent mechanical properties, and reproducibility. The authors will be studying the interfacial properties between epoxy resin and GO-PANI more deeply through molecular dynamics simulations because there are limitations in experimental methods to investigate interfacial properties. After all, the scale is too small to observe.

## Figures and Tables

**Table 1:** The molar ratio of the SV-Neat and DSV-Neat.

Sample Code	Epoxy Resin			Hardener
	DGEBA	PPGDG	DGCHD	2-AFD
SV-Neat	1	0.5	1	1.69
DSV-Neat	1	0.6	1	1.77

**Table 2:** The amount of the SV and DSV resin, and GO-PANI in the nanocomposites.

Sample Code	SV Resin (g)	GO-PANI (g)
SV-Neat	11.98	-
SV-GPN0.5	11.98	0.006
SV-GPN1	11.98	0.012
SV-GPN2	11.98	0.024
DSV-GPN1	11.98	0.012

**Table 3:** Thermal stability of SV-Neat, SV-GPN0.5, SV-GPN1, and SV-GPN2.

Sample Code	T_5%_ (°C)	T_30%_ (°C)	T_dmax_ (°C)	T_s_ (°C)
SV-Neat	253.7	312.2	332.4	141.5
SV-GPN0.5	252.4	310.3	339.5	140.7
SV-GPN1	254.6	314.4	339.1	142.3
SV-GPN2	253.3	310.6	339.3	141.0

## Data Availability

Raw data are deposited at Seoul National University. The original contributions presented in the study are included in the article/Supplementary Materials, further inquiries can be directed to the corresponding author.

## Supplementary Materials

The following supporting information can be downloaded at https://www.mdpi.com/article/10.3390/polym16223173/s1, Figure S1: The structural formula of starting materials corresponding to (a) epoxy resin and (b) diamine, Figure S2: Photograph of GPN dispersed in epoxy resin of (a) SV-GPN0.5, (b) SV-GPN1 and (c) SV-GPN2. Note that the sample did not include AFD hardener, Figure S3: FTIR graph of (a) SV-Neat, (b) SV-GPN0.5, (c) SV-GPN1, and (d) SV-GPN2 before curing (black line) and after curing (red line), Figure S4: TGA graph of (a) SV, (b) SV-GPN0.5, (c) SV-GPN1 and (d) SV-GPN2, Figure S5: FTIR spectra of (a) SV-Neat, (b) SV-GPN0.5, (c) SV-GPN1, and (d) SV-GPN2, Figure S6: Schematic illustration of thermal expansion difference between epoxy resin and GO-PANI filler. After heating, the tiny gap remains, which causes the resin to grip the filler weakly, Figure S7: Stress-strain curve of (a) DSV-GPN1 and (b) healed DSV-GPN1. Table S1: Calculating degree of curing based on the area under FTIR curve.

## References

[1] Kanu N.J., Gupta E., Vates U.K., Singh G.K. (2019). Self-healing composites: A state-of-the-art review. Compos. Part A Appl. Sci. Manuf..

[2] Wang S., Urban M.W. (2020). Self-healing polymers. Nat. Rev. Mater..

[3] Oh J.Y., Son D., Katsumata T., Lee Y., Kim Y., Lopez J., Wu H.-C., Kang J., Park J., Gu X. (2019). Stretchable self-healable semiconducting polymer film for active-matrix strain-sensing array. Sci. Adv..

[4] Kang J., Tok J.B.-H., Bao Z. (2019). Self-healing soft electronics. Nat. Electron..

[5] Cai G., Wang J., Qian K., Chen J., Li S., Lee P.S. (2017). Extremely stretchable strain sensors based on conductive self-healing dynamic cross-links hydrogels for human-motion detection. Adv. Sci..

[6] Liu M., Zhu S., Huang Y., Lin Z., Liu W., Yang L., Ge D. (2021). A self-healing composite actuator for multifunctional soft robot via photo-welding. Compos. Part B Eng..

[7] Acome E., Mitchell S.K., Morrissey T., Emmett M., Benjamin C., King M., Radakovitz M., Keplinger C. (2018). Hydraulically amplified self-healing electrostatic actuators with muscle-like performance. Science.

[8] Xu J., Gao F., Wang H., Dai R., Dong S., Wang H. (2023). Organic/inorganic hybrid waterborne polyurethane coatings with self-healing properties for anticorrosion application. Prog. Org. Coat..

[9] Li B., Xue S., Mu P., Li J. (2022). Robust self-healing graphene oxide-based superhydrophobic coatings for efficient corrosion protection of magnesium alloys. ACS Appl. Mater. Interfaces.

[10] Kumar M.H., Moganapriya C., Kumar A.M., Rajasekar R., Gobinath V. (2021). Self-Healing Materials in Aerospace Applications. Self-Healing Smart Materials and Allied Applications.

[11] Das R., Melchior C., Karumbaiah K. (2016). Self-healing composites for aerospace applications. Advanced Composite Materials for Aerospace Engineering.

[12] Das A., Sallat A., Böhme F., Suckow M., Basu D., Wießner S., Stöckelhuber K.W., Voit B., Heinrich G. (2015). Ionic modification turns commercial rubber into a self-healing material. ACS Appl. Mater. Interfaces.

[13] Wang H., Liu H., Cao Z., Li W., Huang X., Zhu Y., Ling F., Xu H., Wu Q., Peng Y. (2020). Room-temperature autonomous self-healing glassy polymers with hyperbranched structure. Proc. Natl. Acad. Sci. USA.

[14] Santana M.H., Huete M., Lameda P., Araujo J., Verdejo R., López-Manchado M.A. (2018). Design of a new generation of sustainable SBR compounds with good trade-off between mechanical properties and self-healing ability. Eur. Polym. J..

[15] Eom Y., Kim S.-M., Lee M., Jeon H., Park J., Lee E.S., Hwang S.Y., Park J., Oh D.X. (2021). Mechano-responsive hydrogen-bonding array of thermoplastic polyurethane elastomer captures both strength and self-healing. Nat. Commun..

[16] Guo Z., Bao C., Wang X., Lu X., Sun H., Li X., Li J., Sun J. (2021). Room-temperature healable, recyclable and mechanically super-strong poly (urea-urethane) s cross-linked with nitrogen-coordinated boroxines. J. Mater. Chem. A.

[17] Wang D., Wang Z., Ren S., Xu J., Wang C., Hu P., Fu J. (2021). Molecular engineering of a colorless, extremely tough, superiorly self-recoverable, and healable poly (urethane–urea) elastomer for impact-resistant applications. Mater. Horiz..

[18] Duan N., Sun Z., Ren Y., Liu Z., Liu L., Yan F. (2020). Imidazolium-based ionic polyurethanes with high toughness, tunable healing efficiency and antibacterial activities. Polym. Chem..

[19] Zhou J., Yang Y., Qin R., Xu M., Sheng Y., Lu X. (2019). Robust poly (urethane-amide) protective film with fast self-healing at room temperature. ACS Appl. Polym. Mater..

[20] Guo Y., An X., Qian X. (2023). Mechanochromic Self-Healing Materials with Good Stretchability, Shape Memory Behavior, Cyclability, and Reversibility Based on Multiple Hydrogen Bonds. ACS Appl. Mater. Interfaces.

[21] Yao Y., Liu B., Xu Z., Yang J., Liu W. (2021). An unparalleled H-bonding and ion-bonding crosslinked waterborne polyurethane with super toughness and unprecedented fracture energy. Mater. Horiz..

[22] Li Y., Li W., Sun A., Jing M., Liu X., Wei L., Wu K., Fu Q. (2021). A self-reinforcing and self-healing elastomer with high strength, unprecedented toughness and room-temperature reparability. Mater. Horiz..

[23] Li Z., Zhu Y.L., Niu W., Yang X., Jiang Z., Lu Z.Y., Liu X., Sun J. (2021). Healable and recyclable elastomers with record-high mechanical robustness, unprecedented crack tolerance, and superhigh elastic restorability. Adv. Mater..

[24] Zhu X., Zhang W., Lu G., Zhao H., Wang L. (2022). Ultrahigh mechanical strength and robust room-temperature self-healing properties of a polyurethane–graphene oxide network resulting from multiple dynamic bonds. Acs Nano.

[25] Shekar R.I., Kotresh T., Rao P.D., Kumar K. (2009). Properties of high modulus PEEK yarns for aerospace applications. J. Appl. Polym. Sci..

[26] Wei L., Zhao X., Yu Q., Zhang W., Zhu G. (2021). In-plane compression behaviors of the auxetic star honeycomb: Experimental and numerical simulation. Aerosp. Sci. Technol..

[27] Guadagno L., Raimondo M., Vittoria V., Vertuccio L., Naddeo C., Russo S., De Vivo B., Lamberti P., Spinelli G., Tucci V. (2014). Development of epoxy mixtures for application in aeronautics and aerospace. Rsc Adv..

[28] Yu S., Yang S., Cho M. (2009). Multi-scale modeling of cross-linked epoxy nanocomposites. Polymer.

[29] Kausar A., Ahmad I., Maaza M., Bocchetta P. (2023). Self-Healing Nanocomposites—Advancements and Aerospace Applications. J. Compos. Sci..

[30] Krishnakumar B., Sanka R.S.P., Binder W.H., Park C., Jung J., Parthasarthy V., Rana S., Yun G.J. (2020). Catalyst free self-healable vitrimer/graphene oxide nanocomposites. Compos. Part B Eng..

[31] Caglayan C., Kim G., Yun G.J. (2022). CNT-Reinforced Self-Healable Epoxy Dynamic Networks Based on Disulfide Bond Exchange. ACS Omega.

[32] Hu Z., Zhang D., Lu F., Yuan W., Xu X., Zhang Q., Liu H., Shao Q., Guo Z., Huang Y. (2018). Multistimuli-responsive intrinsic self-healing epoxy resin constructed by host–guest interactions. Macromolecules.

[33] Li Z.-J., Zhong J., Liu M.-C., Rong J.-C., Yang K., Zhou J.-Y., Shen L., Gao F., He H.-F. (2020). Investigation on self-healing property of epoxy resins based on disulfide dynamic links. Chin. J. Polym. Sci..

[34] Li B., Zhu G., Hao Y., Ren T. (2022). An investigation on the performance of epoxy vitrimers based on disulfide bond. J. Appl. Polym. Sci..

[35] Lu L., Fan J., Li G. (2016). Intrinsic healable and recyclable thermoset epoxy based on shape memory effect and transesterification reaction. Polymer.

[36] Sun W., Luo J., Zhang L., Liang Y., Chen Y., Zhou H., Zheng Y., Cheng Y. (2022). Healable epoxy-based dielectric semi-interpenetrating networks with different degrees of cross-linking. J. Appl. Polym. Sci..

[37] Li G., Zhang P., Huo S., Fu Y., Chen L., Wu Y., Zhang Y., Chen M., Zhao X., Song P. (2021). Mechanically strong, thermally healable, and recyclable epoxy vitrimers enabled by ZnAl-layer double hydroxides. ACS Sustain. Chem. Eng..

[38] Huang Q.-S., Zhao P.-C., Lai J.-C., Zhang X.-P., Li C.-H. (2022). A healable, recyclable and thermochromic epoxy resin for thermally responsive smart windows. Polym. Chem..

[39] Chen T., Fang L., Li X., Gao D., Lu C., Xu Z. (2020). Self-healing polymer coatings of polyurea-urethane/epoxy blends with reversible and dynamic bonds. Prog. Org. Coat..

[40] Hu Z., Liu Y., Xu X., Yuan W., Yang L., Shao Q., Guo Z., Ding T., Huang Y. (2019). Efficient intrinsic self-healing epoxy acrylate formed from host-guest chemistry. Polymer.

[41] Behera P.K., Raut S.K., Mondal P., Sarkar S., Singha N.K. (2021). Self-healable polyurethane elastomer based on dual dynamic covalent chemistry using Diels–Alder “click” and disulfide metathesis reactions. ACS Appl. Polym. Mater..

[42] Sun W., Zhang L., Liang Y., Xu J., Gao Y., Luo J., Cheng Y. (2022). Mechanically robust epoxy with electrical breakdown healing capability for power equipment insulation via dynamic networks. React. Funct. Polym..

[43] Yuan D., Delpierre S.b., Ke K., Raquez J.-M., Dubois P., Manas-Zloczower I. (2019). Biomimetic water-responsive self-healing epoxy with tunable properties. ACS Appl. Mater. Interfaces.

[44] Zhu K., Li Z., Cheng F., Wu C., Cai D., Zhang Q., Zhang H. (2021). Preparation of durable superhydrophobic composite coatings with photothermal conversion precisely targeted configuration self-healability and great degradability. Compos. Sci. Technol..

[45] Abdul Jaleel S.A., Kim T., Baik S. (2023). Covalently Functionalized Leakage-Free Healable Phase-Change Interface Materials with Extraordinary High-Thermal Conductivity and Low-Thermal Resistance. Adv. Mater..

[46] Liguori A., Subramaniyan S., Yao J.G., Hakkarainen M. (2022). Photocurable extended vanillin-based resin for mechanically and chemically recyclable, self-healable and digital light processing 3D printable thermosets. Eur. Polym. J..

[47] Qin J., Liu X., Chen B., Liu J., Wu M., Tan L., Yang C., Liang L. (2022). Thermo-healing and recyclable epoxy thermosets based on dynamic phenol-carbamate bonds. React. Funct. Polym..

[48] Putnam-Neeb A.A., Kaiser J.M., Hubbard A.M., Street D.P., Dickerson M.B., Nepal D., Baldwin L.A. (2022). Self-healing and polymer welding of soft and stiff epoxy thermosets via silanolates. Adv. Compos. Hybrid Mater..

[49] Xu J., Chen J., Zhang Y., Liu T., Fu J. (2021). A fast room-temperature self-healing glassy polyurethane. Angew. Chem. Int. Ed..

[50] Hodgkin J., Simon G.P., Varley R.J. (1998). Thermoplastic toughening of epoxy resins: A critical review. Polym. Adv. Technol..

[51] Xiao Z.-T., Wu G.-L., Wang W., Zhang P., Hu Y., Wang X. (2024). Novel phosphorous-containing epoxy thermosets with improved anti-flammability, smoke suppression, and dielectric properties. Polym. Degrad. Stab..

[52] Konstantinova A., Yudaev P., Shapagin A., Panfilova D., Palamarchuk A., Chistyakov E. (2024). Non-Flammable Epoxy Composition Based on Epoxy Resin DER-331 and 4-(β-Carboxyethenyl) phenoxy-phenoxycyclotriphosphazenes with Increased Adhesion to Metals. Sci.

[53] Kadam A., Pawar M., Yemul O., Thamke V., Kodam K. (2015). Biodegradable biobased epoxy resin from karanja oil. Polymer.

[54] Yao T., Zhang C., Chen K., Niu T., Wang J., Yang Y. (2023). Hydroxyl-group decreased dielectric loss coupled with 3D-BN network enhanced high thermal conductivity epoxy composite for high voltage-high frequency conditions. Compos. Sci. Technol..

[55] Wu Y., Fan X., Wang Z., Zhang Z., Liu Z. (2024). A mini-review of ultra-low dielectric constant intrinsic epoxy resins: Mechanism, preparation and application. Polym. Adv. Technol..

[56] Kim G., Caglayan C., Yun G.J. (2022). Epoxy-Based Catalyst-Free Self-Healing Elastomers at Room Temperature Employing Aromatic Disulfide and Hydrogen Bonds. ACS Omega.

[57] Kim B., Youn B., Song Y., Lee D. (2022). Enhanced dispersion stability and interfacial damping of POSS-functionalized graphene oxide in PDMS nanocomposites. Funct. Compos. Struct..

[58] Proscovia K., Cheon H.J., Shin S.Y., Jeong G., Go S., Kim K.S., Park R.-S., Huh Y.-I., Chang M. (2022). Enhanced charge transport of conjugated polymer/reduced graphene oxide composite films by solvent vapor pre-treatment. Funct. Compos. Struct..

[59] Harsha V.S.S., Sharma A., Tambe P. (2022). Graphene oxide reinforced epoxy nanocomposites coatings for corrosion protection: A review. J. Phys. Conf. Ser..

[60] Yarahmadi E., Didehban K., Sari M.G., Saeb M.R., Shabanian M., Aryanasab F., Zarrintaj P., Paran S.M.R., Mozafari M., Rallini M. (2018). Development and curing potential of epoxy/starch-functionalized graphene oxide nanocomposite coatings. Prog. Org. Coat.

[61] Ferreira H., Poma G., Acosta D., Barzola-Quiquia J., Quintana M., Barreto L., Champi A. (2018). Laser power influence on Raman spectra of multilayer graphene, multilayer graphene oxide and reduced multilayer graphene oxide. J. Phys. Conf. Ser..

[62] Ge G., Lu Y., Qu X., Zhao W., Ren Y., Wang W., Wang Q., Huang W., Dong X. (2019). Muscle-inspired self-healing hydrogels for strain and temperature sensor. ACS Nano.

[63] He D., Peng Z., Gong W., Luo Y., Zhao P., Kong L. (2015). Mechanism of a green graphene oxide reduction with reusable potassium carbonate. RSC Adv..

[64] Zhang P., Han X., Kang L., Qiang R., Liu W., Du Y. (2013). Synthesis and characterization of polyaniline nanoparticles with enhanced microwave absorption. Rsc Adv..

[65] Krishnamoorthy K., Veerapandian M., Yun K., Kim S.-J. (2013). The chemical and structural analysis of graphene oxide with different degrees of oxidation. Carbon.

[66] Lin-Vien D., Colthup N.B., Fateley W.G., Grasselli J.G. (1991). The Handbook of Infrared and Raman Characteristic Frequencies of Organic Molecules.

[67] Abdullah H.S. (2012). Electrochemical polymerization and Raman study of polypyrrole and polyaniline thin films. Int. J. Phys. Sci.

[68] Song P., Zhang X., Sun M., Cui X., Lin Y. (2012). Synthesis of graphene nanosheets via oxalic acid-induced chemical reduction of exfoliated graphite oxide. Rsc. Adv..

[69] Amrollahi S., Ramezanzadeh B., Yari H., Ramezanzadeh M., Mahdavian M. (2019). Synthesis of polyaniline-modified graphene oxide for obtaining a high performance epoxy nanocomposite film with excellent UV blocking/anti-oxidant/anti-corrosion capabilities. Compos. Part B Eng..

[70] Murali A., Sampath S., Appukutti Achuthan B., Sakar M., Chandrasekaran S., Suthanthira Vanitha N., Joseph Bensingh R., Abdul Kader M., Jaisankar S.N. (2020). Copper (0) mediated single electron transfer-living radical polymerization of methyl methacrylate: Functionalized graphene as a convenient tool for radical initiator. Polymers.

[71] Dhamodharan D., Dhinakaran V., Ghoderao P.N., Byun H.-S., Wu L. (2022). Synergistic effect of cellulose nanocrystals-graphene oxide as an effective nanofiller for enhancing properties of solventless polymer nanocomposites. Compos. Part B Eng..

[72] Nonahal M., Rastin H., Saeb M.R., Sari M.G., Moghadam M.H., Zarrintaj P., Ramezanzadeh B. (2018). Epoxy/PAMAM dendrimer-modified graphene oxide nanocomposite coatings: Nonisothermal cure kinetics study. Prog. Org. Coat..

[73] Dinu R., Lafont U., Damiano O., Mija A. (2022). High glass transition materials from sustainable epoxy resins with potential applications in the aerospace and space sectors. ACS Appl. Polym. Mater..

[74] Shi G., Song J., Tian X., Liu T., Wu Z. (2024). High-performance epoxy nanocomposites via constructing a rigid-flexible interface with graphene oxide functionalized by polyetheramine and f-SiO_2_. Carbon.

[75] Zheng W., Angelopoulos M., Epstein A.J., MacDiarmid A. (1997). Experimental evidence for hydrogen bonding in polyaniline: Mechanism of aggregate formation and dependency on oxidation state. Macromolecules.

[76] Prusty R.K., Rathore D.K., Sahoo S., Parida V., Ray B.C. (2017). Mechanical behaviour of graphene oxide embedded epoxy nanocomposite at sub-and above-zero temperature environments. Compos. Commun..

