# Supplementary material for: High Modulus Epoxy/GO-PANI Self-Healing Materials Without Catalyst by Molecular Engineering and Nanocomposite Fabrication
Source: Polymers (Basel). 2024 Nov 14;16(22):3173. doi: 10.3390/polym16223173 (PMC11598404; doi:10.3390/polym16223173)
Supplement: Supplementary file 1 [file polymers-16-03173-s001.zip › polymers-3246551-supplementary.pdf]

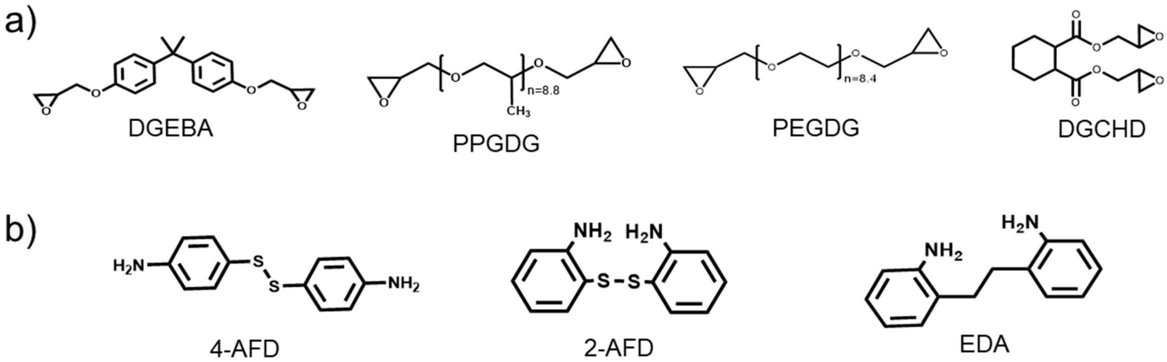

Figure S1. The structural formula of starting materials corresponding to a) epoxy resin and b) diamine.

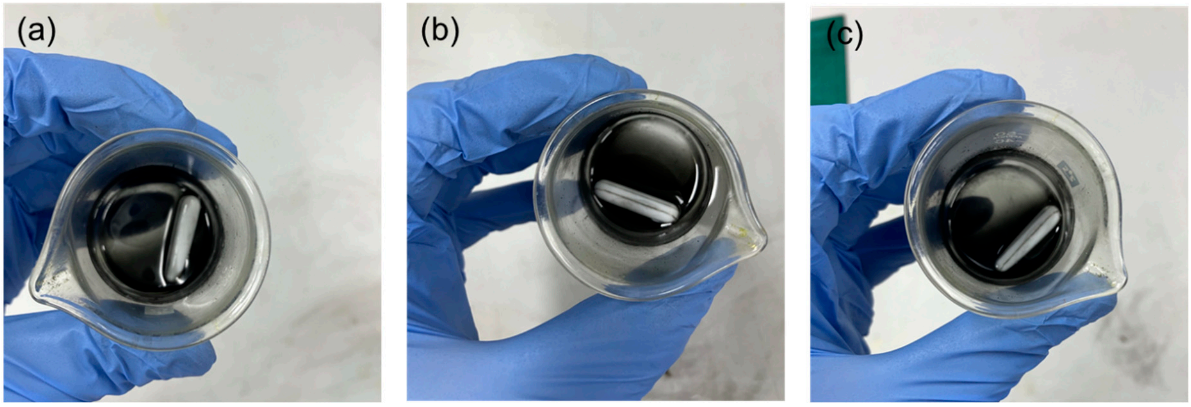

Figure S2. Photograph of GPN dispersed in epoxy resin of (a) SV-GPN0.5, (b) SV-GPN1 and (c) SV-GPN2. Note that the sample did not include AFD hardener.

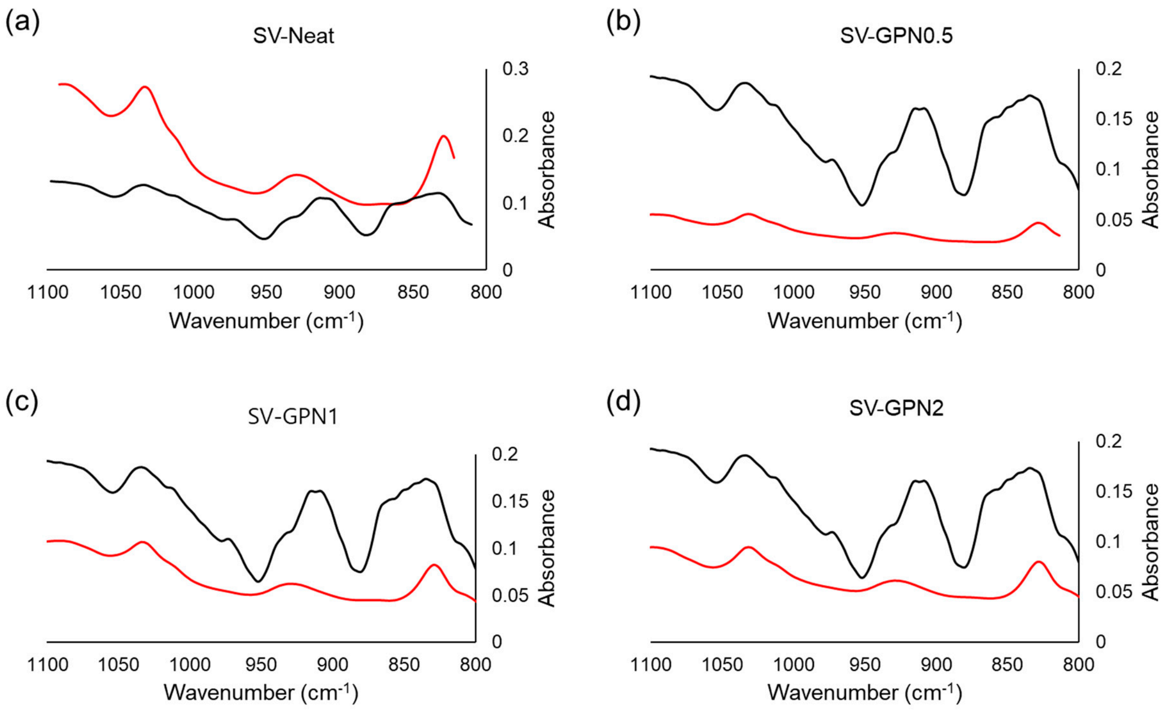

Figure S3. FTIR graph of (a) SV-Neat, (b) SV-GPN0.5, (c) SV-GPN1, and (d) SV-GPN2 before curing (black line) and after curing (red line).

Table S1. Calculating degree of curing based on the area under FTIR curve

Sample	h_{before}^{epoxy} [a]	h_{before}^{ether} [b]	h_{after}^{epoxy} [c]	h_{after}^{ether} [d]	Degree of curing (%) [e]
**SV-PRI**	0.053486	0.0288076	0.033989	0.0710479	74.2
**SV-GPN0.5**	0.089550	0.0472532	0.006504	0.0126590	72.9
**SV-GPN1**	0.089550	0.0472532	0.013707	0.0245995	71.0
**SV-GPN2**	0.089550	0.0472532	0.012496	0.0262070	74.8

[a] h_{before}^{epoxy} is the height of the epoxide ring peak before the curing reaction. [b] h_{before}^{ether} is the height of the ether bond before the curing reaction. [c] h_{after}^{epoxy} is the height of the epoxide ring after the curing reaction. [d] h_{after}^{ether} is the height of the ether bond after the curing reaction. [e] Degree of curing is calculated through Eq. (2). FTIR graph is drawn in Figure S2.

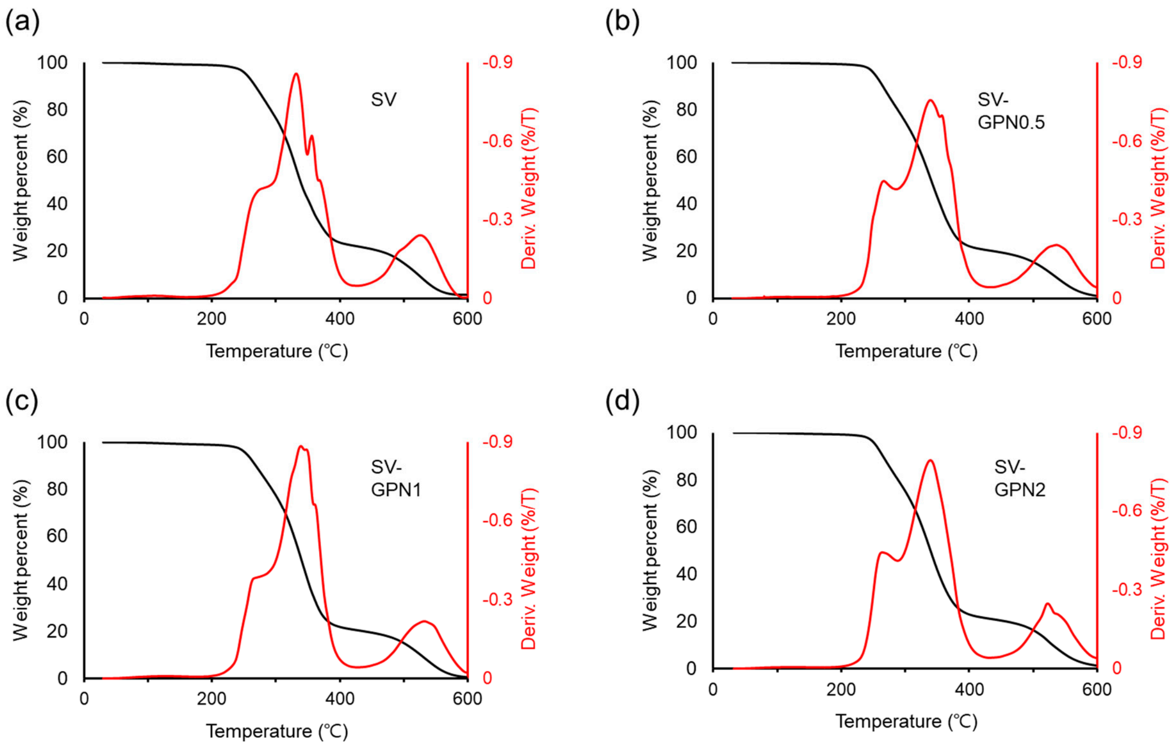

Figure S4. TGA graph of (a) SV, (b) SV-GPN0.5, (c) SV-GPN1 and (d) SV-GPN2.

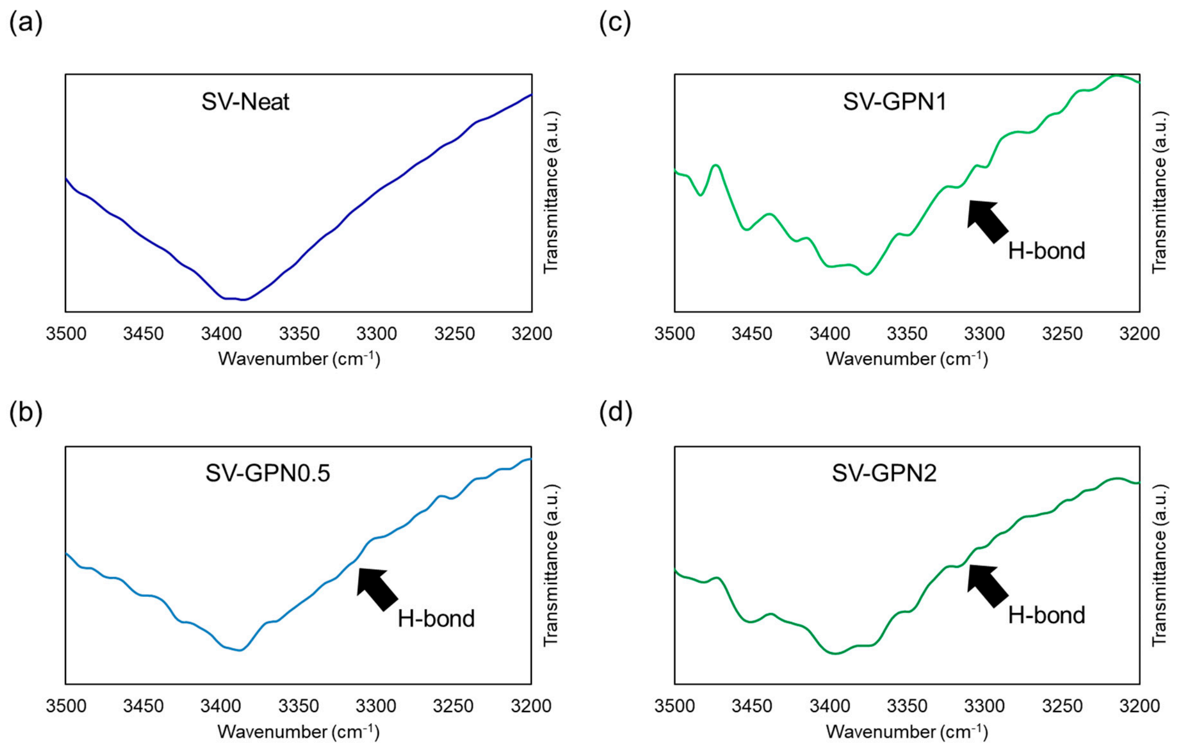

Figure S5. FTIR spectra of (a) SV-Neat, (b) SV-GPN0.5, (c) SV-GPN1, and (d) SV-GPN2

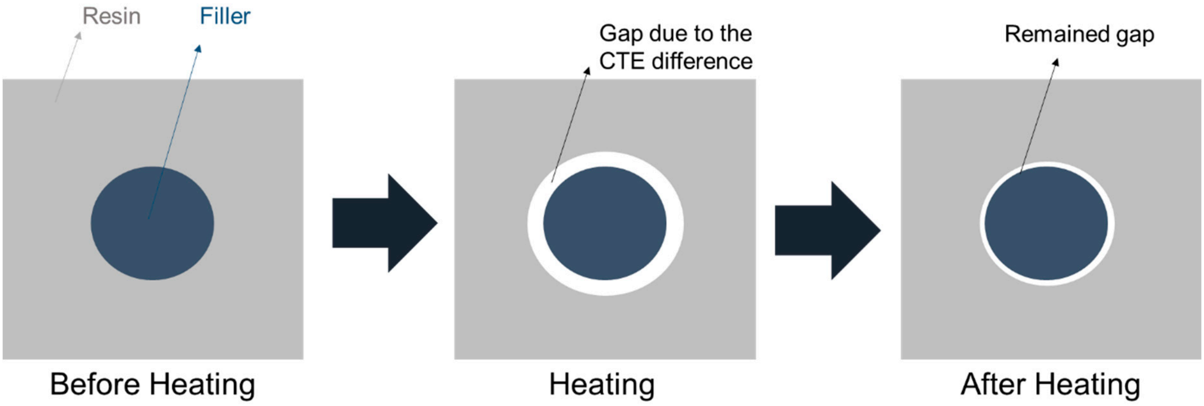

Figure S6. Schematic illustration of thermal expansion difference between epoxy resin and GO-PANI filler. After heating, the tiny gap remains, which causes the resin to grip the filler weakly.

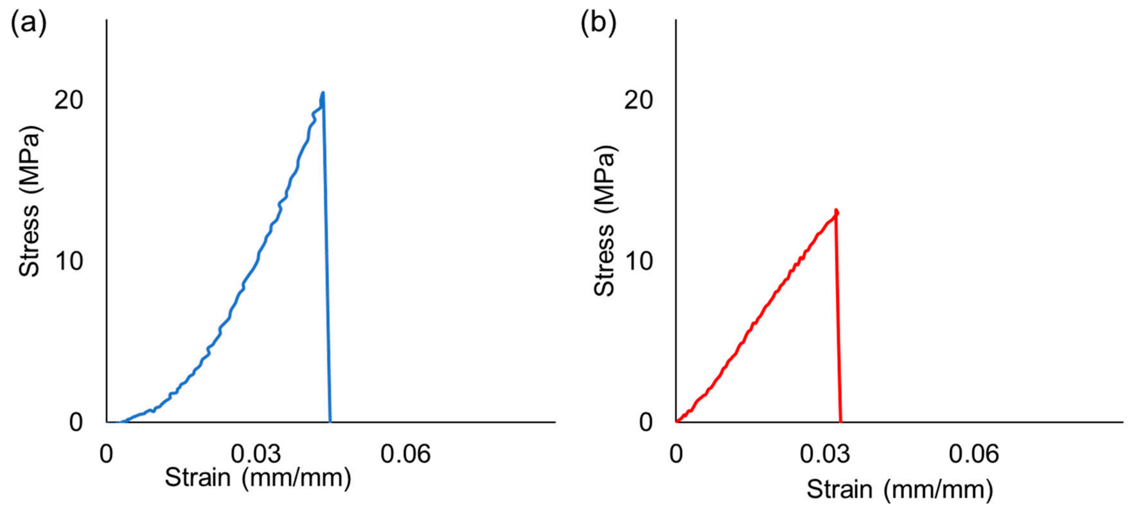

Figure S7. Stress-strain curve of (a) DSV-GPN1 and (b) healed DSV-GPN1.
